# *Bacillus amyloliquefaciens* BA5 Attenuates Carbon Tetrachloride-Induced Hepatotoxicity in Mice

**DOI:** 10.3390/nu18020298

**Published:** 2026-01-17

**Authors:** Yuanyuan He, Feiran Li, Yangrui Li, Mengen Xu, Chuxian Quan, Shah Nawaz, Md. F. Kulyar, Mudassar Iqbal, Jiakui Li

**Affiliations:** 1 College of Veterinary Medicine, Huazhong Agricultural University, Wuhan 430070, China; hyy229@webmail.hzau.edu.cn (Y.H.); lfr1997@webmail.hzau.edu.cn (F.L.); liyangrui@webmail.hzau.edu.cn (Y.L.); xme183xme@163.com (M.X.); qcx2000@webmail.hzau.edu.cn (C.Q.); mudassar.iqbal@iub.edu.pk (M.I.); 2 Department of Regenerative Medicine, State Research Institute Centre for Innovative Medicine, LT-08406 Vilnius, Lithuania; fakharealam786@hotmail.com; 3 College of Animals Husbandry and Veterinary Medicine, Tibet Agricultural and Animal Husbandry University, Linzhi 860000, China

**Keywords:** *Bacillus amyloliquefaciens*, CCl_4_, hepatoprotective effects, oxidative stress, gut microbiota

## Abstract

**Background:** The association between liver disease and gut microbiota is being widely investigated. Probiotics, such as *Bacillus amyloliquefaciens*, are among the most notable microbiomes examined in this study. *Bacillus amyloliquefaciens* shows potential for promoting growth and effectively regulating gut microbiota, though its mechanism of action remains unclear. **Methods:** The early gavage administration of *Bacillus amyloliquefaciens* BA5 conferred protection against liver injury in carbon tetrachloride (CCl_4_)-induced mice. Growth parameters (body weight and organ index), serum biochemical markers (ALT, AST, T-SOD, MDA, GSH-Px, and T-AOC), liver and jejunum histopathology, and gut microbiota composition were comprehensively evaluated. **Results:** BA5 supplementation restored serum T-AOC, T-SOD, and GSH-Px levels and attenuated CCl_4_-induced increases in ALT, AST, and MDA, suggesting potent anti-oxidant properties. Furthermore, histopathologic assessment showed that CCl_4_-induced mice developed acute liver injury and intestinal villi were destroyed, while the BA5 group restored the pathological changes in the tissues to the normal group level. In addition, immunohistochemical staining revealed that BA5 increased the expression level of Claudin-1 which was a key biomarker for assessing the integrity of epithelial/endothelial barriers. Regarding gut microbiota, BA5 significantly enhanced the abundance of beneficial bacteria (*Lactobacillus*) and decreased the abundance of hazardous bacteria (*Fusobacterium*, *Lachnoclostridium*, *Phascolarctobacterium*, and *Escherichia–shigella*) caused by CCl_4_. Notably, BA5 alone remarkably increased gut microbial diversity compared with that of the Control group. **Conclusions:** Overall, these findings suggest that BA5 holds promise as a potential therapeutic agent for alleviating CCl_4_-induced acute liver injury in mice by mitigating oxidative stress and modulating gut microbiota.

## 1. Introduction

Liver diseases cause significant morbidity and mortality worldwide, being responsible for approximately 2,000,000 deaths worldwide per year [1,2]. This issue presents a worldwide medical challenge because the liver is the principal detoxification organ. The liver metabolizes multiple compounds, which produce reactive oxygen radicals [3]. These radicals mainly include hydrogen peroxide, hydroxyl radicals, and superoxide anions. Such species contribute to the reactivity of various targets involved in both physiological and pathological processes. Oxidative stress is defined as an imbalance between pro-oxidants and anti-oxidants. This imbalance leads to extensive histopathological lesions that can range from mild hepatitis to hepatocellular carcinoma [4]. It is known as a mechanism that causes cell cytotoxicity, and excessive oxidative stress is responsible for DNA damage and cell apoptosis [5]. Furthermore, the pathogenesis of many degenerative diseases, such as diabetes, cardiovascular disorders, and neurodegenerative diseases, is closely linked to oxidative stress [6]. Although mammals possess well-developed anti-oxidant systems to maintain redox biology, e.g., enzymes and non-enzyme anti-oxidants, the abundance of toxic substances with pro-oxidant properties can still cause severe hepatotoxicity.

Growing evidence demonstrates that crosstalk between the gut microbiota, its derived metabolites, and the liver plays a key role in the pathogenesis of many liver diseases, including chronic hepatitis B [7], non-alcoholic steatohepatitis [8], and non-alcoholic fatty liver disease [9]. The gut–liver axis results from both the functional and anatomical, bidirectional interaction of the liver and gastrointestinal tract, and this interaction is built through a portal circulation, which carries products derived from the gut to the liver, and the feedback of molecules, e.g., antibodies and bile, from the liver to the gut [10]. The gut microbiome is a diverse ecosystem that consists of trillions of microorganisms, which exist in a specific symbiosis with the host body [11]. The gut microbiome plays an indispensable role in both physiological and pathological aspects of host health, contributing to key processes such as digestion and immunomodulation. It is increasingly recognized as a ‘hidden metabolic organ’ due to its extensive functional capabilities. Disturbance of the gut microbiota leads to dysbiosis, which increases gut permeability and allows microorganisms and pathogen-associated molecular patterns (PAMPs) to translocate [12]. When these PAMPs (e.g., endotoxin) and microbiome-derived metabolites cross the dysfunctional gut barrier and penetrate the liver via the portal influx, they trigger inflammation cascades that worsens hepatic inflammation [13]. Conversely, the progression of liver diseases impacts the gut through the systemic circulation and biliary system by releasing inflammatory mediators and bile acids, further impairing the gut barrier [14]. Hence, liver disease progression connects closely to the severity of gut dysbiosis, which is supported by previous research, i.e., a specific microbiota pattern has been recognized in the gut of patients with serious alcohol hepatitis [15]. The control of gut microbiota is important for maintaining the homeostasis of the gut–liver axis in a host. Beyond antibiotics, upcoming therapies of liver diseases focus on the gut, including probiotics, fecal microbial transplantation, bacterial metabolites, and carbon nanoparticles [16].

In recent years, probiotics have emerged as a promising therapeutic alternative to antibiotics, aiming to reduce feed-associated organ toxicity in the host [17]. Prior studies have shown that probiotics possess beneficial impacts for the host’s health, including the regulation of the gut microbiome, the stimulation of the immune protection mechanism response, and the production of anti-microbial substances, etc., as a promising feed additive [18]. *Bacillus amyloliquefaciens* (*B. amyloliquefaciens*), a probiotic species that can be administrated directly to animals, has a high stability under harsh conditions such as moisture, heat, and gastric environments [19]. By stabilizing the gut microbiota balance and secreting enzymes such as β-glucanase, amylase, and xylanase, it enhances nutrient absorption, feed efficiency, and meat quality [20,21]. Currently, *Bacillus amyloliquefaciens* is also being investigated for its role in microbial detoxification, including the reduction in Aflatoxin B1 residues, the modulation of gut microbial balance, the enhancement of anti-oxidant enzyme activities, and the mitigation of liver oxidative damage [22,23]. Our previous study demonstrated that *B. amyloliquefaciens* (BA5) has good potential probiotic effects in in vivo and in vitro experiments [24]. However, whether *B. amyloliquefaciens* BA5 plays hepatoprotective effects in mice remains unclear, as well as the mechanism of action.

Therefore, it was hypothesized that *B. amyloliquefaciens* BA5 would confer hepatoprotective effects by modulating the gut microbiome and attenuating intestinal injury in a liver injury murine model. In the current experiment, a CCl_4_-induced liver injury model was used. This compound has been widely used to induce hepatic injury in various animal models to investigate the underlying mechanisms of hepatotoxicity and liver damage [25]. Given that alterations in the gut–liver axis play a crucial role in the pathogenesis of liver diseases, identifying the disrupted nodes within this axis offers promising avenues for future probiotic-based therapeutic strategies.

## 2. Materials and Methods

### 2.1. Reagents

For animal experiments, CCl_4_ (10006464) and olive oil (69018028) were purchased from Reagen (Shanghai, China). The *B. amyloliquefaciens* strain (BA5) used in the current study originated from our previous study [24].

### 2.2. Animal Experiments

A total of 40 male Kunming mice (18–20 g, six weeks old) [26,27] were purchased from the Laboratory Animal Center of Huazhong Agricultural University (Wuhan, China). Mice were housed under a 12 h light/dark cycle at 24 ± 2 °C and 50–55% humidity, with free access to standard rodent chow and water. After one week of acclimatization, mice were randomly assigned to control (Control), probiotic (BA5), model (CCl_4_), and probiotic + CCl_4_ (CCl_4_ + BA5) groups, with 10 mice in each group. From days 8 to 27, BA5 and treatment groups received daily gavage of *B. amyloliquefaciens* BA5 (2 × 10^9^ CFU/mL, 0.5 mL) [28], while Control and CCl_4_ groups received saline.

On day 28, mice in the CCl_4_ and treatment groups were intraperitoneally injected with 1% CCl_4_ olive oil solution (0.01 mL/g·bw, diluted in olive oil), and mice in the Control and BA5 groups were intraperitoneally injected with the same volume of olive oil only. The gavage dose of *B. amyloliquefaciens* BA5 and intraperitoneal injection dose of CCl_4_ in the current experiment were chosen based on previous published test results [24,29]. The body weight of mice was measured and recorded daily prior to gavage. During the test period, all mice were observed and their clinical symptoms were recorded, which included general behavioral activity and clinical signs. All mice were euthanized at 16 h after CCl_4_ olive oil solution or olive oil treatment. Mice were deeply anesthetized with drugs, and upon confirmation of the absence of pedal reflexes, were euthanized by exsanguination via cardiac puncture followed by cervical dislocation, consistent with international ethical guidelines. Anticoagulant-free disposable blood collection tubes were used to collect the blood sample, and the liver and spleen were collected and weighed. Subsequently, their liver and jejunum tissues were placed into aliquots for further histopathological examination and RNA isolation (snap-frozen). The cecal content was placed in liquid nitrogen after collection and subsequently stored at −80 °C for additional study. Throughout the entire experiment, no animals were excluded from the study. All animals entered into the experiment completed the study protocol, and all collected data points were included in the final analysis. The animal experiment was permitted and guided by the Huazhong Agricultural University under the Laboratory Animal Care Ethics Committee (HZAUMO-2023-0226).

### 2.3. Biochemical Parameters Examination in Serum

Blood samples were centrifuged at 3500 rpm/min for 15 min at 4 °C. All serum samples were then stored at −20 °C for hematology analysis. According to the manufacturer’s instructions, the commercial biochemical assay kits (Nanjing Jiancheng Co., Ltd., Nanjing, China) were used to assess the level of oxidative stress and liver injury in mice.

### 2.4. Histopathological Assessment

Liver and jejunum tissues were first fixed in 4% paraformaldehyde fixative, dehydrated in different concentration gradients of alcohol, and then embedded in paraffin. Afterward, the paraffin tissue was sectioned with a thickness of approximately 4 μm and stained with hematoxylin–eosin (H&E). Finally, specimens were observed and described by a pathologist under an optical microscope, and representative images were taken.

The severity of the liver injury was graded on a scale of 1 to 5. The grading depended on the degree of cellular necrosis, coagulum, the central area, and surrounding inflammatory infiltrate. A score of 0 was labeled normal. A score of 1 was labeled very low (<1%). A score of 2 was labeled mild (1–25%). A score of 3 was labeled moderate (26–50%). A score of 4 was labeled moderate/severe (51–75%). A score of 5 was labeled severe/high (76–100%) [30]. The injury score was averaged for each group of animals.

### 2.5. Immunofluorescence Staining

The paraffin-embedded sections of jejunum tissues from the histopathological evaluations (above) were used for immunohistochemical experiments. First, the sections were sequentially immersed in Xylene 1 (15 min), then Xylene 2 (15 min), followed by ethanol 1 (5 min), ethanol 2 (5 min), 95% ethanol (5 min), 85% ethanol (5 min), and 75% ethanol (5 min). Next, microwave antigen retrieval was performed for 15 min in citrate buffer. After this, nonspecific antigens were blocked with 5% goat serum for 30 min at 37 °C. Then, the sections were incubated with Claudin-1 polyclonal antibody (Proteintech Group, 13050-1-AP, dilution ratio 1:500) at 4 °C overnight, followed by 1 h incubation with HRP-conjugated goat anti-rabbit secondary antibody. The sections were stained with DAPI for 5 min at room temperature, rinsed three times (5 min each) in PBS, and mounted using anti-fluorescence medium and a coverslip. Finally, the prepared fluorescent slide was stored at 4 °C away from light.

### 2.6. Gene Expression Levels Detection Using RT-qPCR

Total RNA was extracted from 100 mg of liver using Trizol reagent (Takara, Beijing, China). RNA concentration and quality were measured with a NanoDrop spectrophotometer (Thermo, Waltham, MA, USA). The RNA was reverse transcribed with a kit (Vazyme, Nanjing, China), then mRNA expression levels of target genes (Table 1) were analyzed by RT-qPCR using a SYBR^®^Green kit (Vazyme, China). Primers for Nrf2, heme oxygenase 1 (HO-1), NAD (P) H: quinone oxidoreductase 1 (NQO1), and glutamate–cysteine ligase catalytic subunit (GCLC) were designed and synthesized by Wuhan Qingke Biotech Bioengineering Co., Ltd. (Wuhan, China). β-actin (Wuhan Qingke Biotech Bioengineering Co., Ltd., China) served as the reference gene.

### 2.7. DNA Extraction

The cecal content samples were subjected to genomic DNA extraction using a TIANamp Genomic DNA kit (TIANGEN Biotechnology Co., Ltd., Beijing, China), according to the manufacturer’s instructions. Genomic DNA was subjected to quality and quantify examination using 1% agarose gel electrophoresis and a NanoDrop spectrophotometer (Thermo Scientific, Waltham, MA, USA).

### 2.8. 16S rDNA Genes Amplification and High-Throughput Sequencing

The V3/V4 hypervariable regions of the 16s rDNA genes were amplified by PCR using sequencing primers with adaptors, which enabled subsequent library construction and sequencing. PCR was performed at 95 °C for 3 min followed by 30 cycles of 95 °C for 30 s, 56 °C for 45 s, and 72 °C for 30 s, with a final elongation step at 72 °C for 10 min. After removing the unspecific products using AGENCOURT AMPure XP beads (Beckman Coulter Commercial Enterprise Co., Ltd., Shanghai, China), the PCR amplicons were evaluated and purified using 1% agarose gel electrophoresis and an AxyPrep DNA Gel Extraction Kit (Axygen, Union City, CA, USA). Subsequently, the purified PCR products were pooled to construct amplicon libraries. Libraries meeting the quality criteria, defined as having a concentration greater than 2 nM and a single peak, were loaded onto the Illumina MiSeq platform (Illumina, San Diego, CA, USA) following the manufacturer’s instructions. Sequencing was carried out using a 2 × 300 bp paired-end configuration.

### 2.9. Bioinformatics and Statistical Analysis

The obtained paired-end reads were merged using FLASH software (v1.2.7), then quality controlled to eliminate chimera using Trimmomatic software (v0.33) and UCHIME software (v4.2). Moreover, short sequences (<200 bp) and low-quality reads were removed. The remaining high-quality sequences were clustered into operational taxonomic units (OTUs) at 97% sequence similarity. Representative bacterial OTU sequences were identified and subjected to phylogenetic analysis using Mothur software (v1.31.2) with reference to the SILVA database. R software (v3.0.3) was used to image Venn diagrams, which could visually display the numbers of unique and common OTUs among groups. Moreover, the corresponding rarefaction curve and rank abundance curve were constituted to evaluate current sequencing depth. Five alpha-diversity indices (Chao1, Shannon, ACE, Simpson, and PD_whole), reflecting species richness and diversity, were calculated using Mothur. Metastats analysis was performed to detect the differentially abundant taxa (phyla and genus) between different groups, while linear discriminant analysis effect size (LEfSe) could analyze the biomarkers with statistical differences. The sample size (*n* = 10 per group) was determined based on similar previously published studies [31,32]. GraphPad Prism (v 7.0) was used to conduct statistical analysis and the T-test was utilized for comparisons between two groups. All values were presented as mean ± SD, and *p*-values (corrected) < 0.05 were considered statistically significant. While the primary study cohort consisted of *n* = 10 mice per group, a subset of *n* = 4 animals per group was randomly selected for histological analysis, immunofluorescence, and intestinal sequencing. Preliminary validation demonstrated that intra-group variability for these endpoints was low; therefore, a sample size of *n* = 4 provided sufficient statistical power to detect significant differences and illustrate the biological effect consistent with the full cohort. To minimize bias, group allocation was blinded during data collection and analysis. Histological samples and raw data files were assigned random numerical codes by an independent investigator. Consequently, the researchers performing the histological scoring and statistical analysis were unaware of the group identities until the analysis was complete.

## 3. Results

### 3.1. B. amyloliquefaciens BA5 Relieved the Dysfunction and Pathological Damage of Livers in CCl_4_-Induced Mice

**Figure 1:**
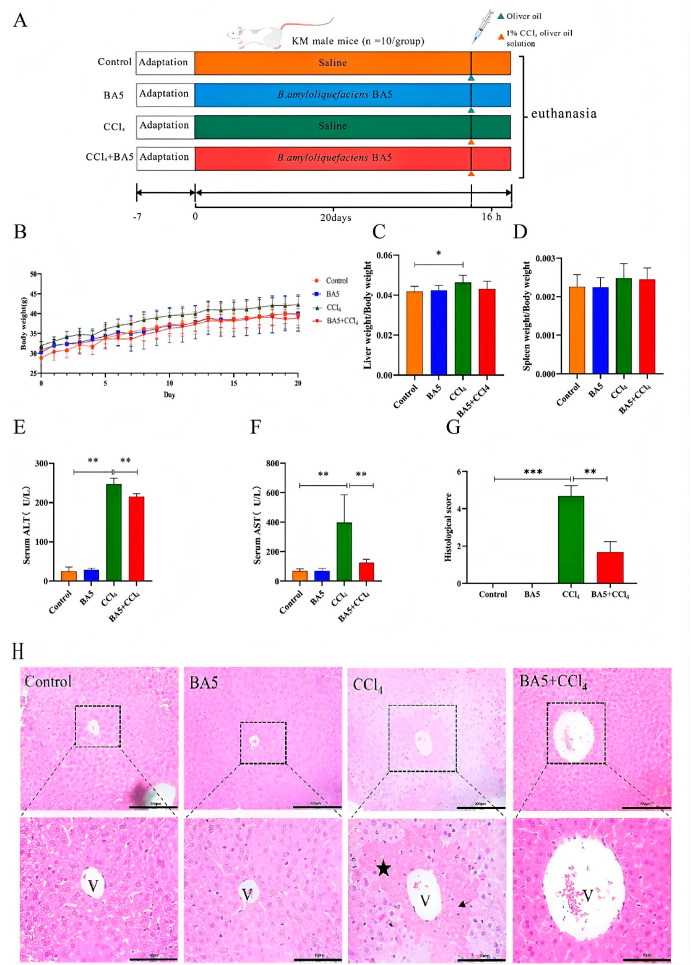
*B. amyloliquefaciens* BA5 alleviated the dysfunction and pathological damage of the liver in CCl_4_-induced mice. (**A**) Animal experiment process. (**B**) Body weight change. (**C**) Liver and (**D**) spleen index. (**E**) ALT and (**F**) AST enzyme activity in serum. (**G**) Histological score. (**H**) H&E pathological section of mice liver tissue. Scale bar: 200 μm and 10 μm, “V” denotes the blood vessel (vascular lumen), and the star marks the corresponding pathological area. Data was expressed as the mean ± SD (*n* = 10; * *p* < 0.05; ** *p* < 0.01; *** *p* < 0.001). the arrow indicates the highlighted histopathological lesion.

Gradually, the body weight of mice increased with longer feeding days, while there was no remarkable difference between different groups (Figure 1B). As shown in Figure 1C, the relative weight of the liver in the CCl_4_ group increased significantly (*p* < 0.05), while there was no remarkable difference in spleen index compared with the Control group.

To evaluate whether BA5 relieved CCl_4_-induced liver dysfunction, serum ALT and AST levels were measured. As shown in Figure 1D,E, CCl_4_ increased ALT and AST, while BA5 significantly reduced (*p* < 0.01) both enzyme activities. Thus, BA5 may counteract CCl_4_’s harmful liver effects. As shown in Figure 1G, no liver abnormalities were seen in the Control and BA5 groups, but the CCl_4_ group showed dramatic discoloration. Hepatocytes were organized and intact in the Control and BA5 groups, while in the CCl_4_ group, they were disorganized, with necrosis (black star) and inflammatory cell infiltration (black arrow). Mice given BA5 before modeling had clearer hepatocyte structures than the CCl_4_ group.

### 3.2. B. amyloliquefaciens BA5 Reduced Oxidative Stress in CCl_4_-Induced Mice

**Figure 2:**
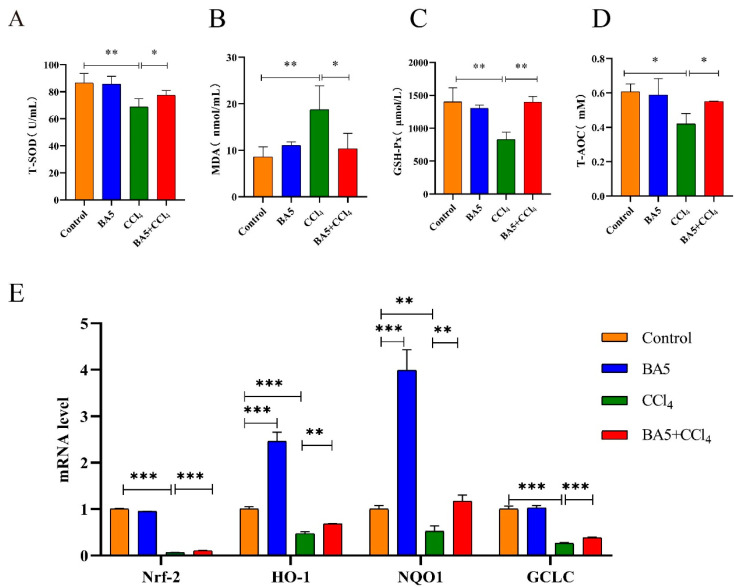
*B. amyloliquefaciens* BA5 reduced oxidative stress in CCl_4_-induced mice. (**A**) The activities of (**A**) T-SOD, (**B**) MDA, (**C**) GSH-Px, (**D**) T-AOC in serum. (**E**) The mRNA expression levels of Nrf2, HO-1, NQO1, and GCLC in liver tissues. Data was expressed as the mean ± SD (*n* = 10; * *p* < 0.05; ** *p* < 0.01; *** *p* < 0.001).

Liver injury was frequently accompanied by oxidative stress. Therefore, the related enzymes were detected (Figure 2A–D). In terms of serum antioxidant-related enzymes and indices, including superoxide dismutase (SOD), glutathione peroxidase (GSH-Px), and total antioxidant capacity (T-AOC), levels were significantly decreased (*p* < 0.05 or *p* < 0.01)In contrast, the level of Malondialdehyde (MDA) significantly improved (*p* < 0.01), suggesting low anti-oxidant activity in the CCl_4_-induced group. In the BA5-treated group, the levels of SOD, GSH-Px, and T-AOC were significantly elevated (*p* < 0.05 or *p* < 0.01), while the level of MDA was significantly reduced (*p* < 0.05) compared with the CCl_4_ group. HO-1, NQO1, and GCLC are important anti-oxidant enzymes. As shown in Figure 2E, the RT-qPCR results suggested that the anti-oxidant-related gene expression of Nrf2, HO-1, NQO1, and GCLC was reduced in the CCl_4_ group compared with the Control group (*p* < 0.01 or 0.001). However, BA5 significantly reversed the reduction in Nrf2, HO-1, NQO1, and GCLC gene expression levels caused by CCl_4_ at the gene levels (*p* < 0.01 or 0.001), indicating that BA5 promoted the activities of anti-oxidant enzymes.

### 3.3. Bacillus amyloliquefaciens BA5 Alleviated Intestinal Injury in CCl_4_-Induced Mice

**Figure 3:**
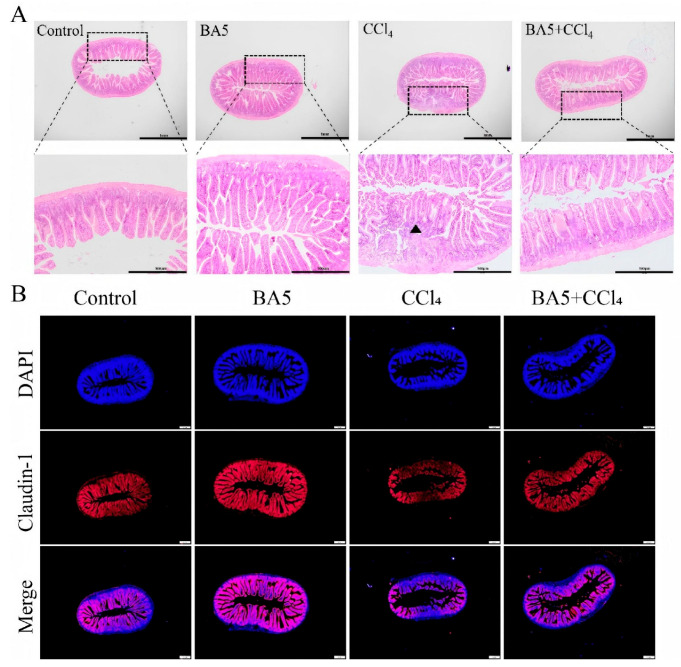
*B. amyloliquefaciens* BA5 relieved liver injury in CCl_4_-induced mice. (**A**) H&E pathological section of mice jejunum tissue. black triangle indicated the highlighted histopathological area/lesion. Scale bar: 1 mm and 500 μm. (**B**) Immunofluorescence staining of the Claudin-1, evidenced by the red = positivity; DAPI (blue) represented cell nuclei. Scale bars = 50 μm. Data are presented as mean ± SD (*n* = 4 biological replicates randomly selected from each group).

In terms of the histopathological observation of the jejunum, H&E staining demonstrated that the jejunum displayed regular and normal structures without obvious pathological changes in the Control group, while the intestinal villi were broken and disorganized with inflammatory cell infiltration (black triangle) in the CCl_4_ group. Interestingly, the BA5 prevention group showed intact and clear intestinal villi compared with the CCl_4_ group (Figure 3A).

Immunohistochemical analysis showed reduced Claudin-1 immunoreactivity in the CCl_4_ group, whereas BA5 treatment significantly restored its expression (Figure 3B).

### 3.4. BA5 Mitigated Gut Microbiota Dysbiosis Induced by CCl_4_ in Mice

**Figure 4:**
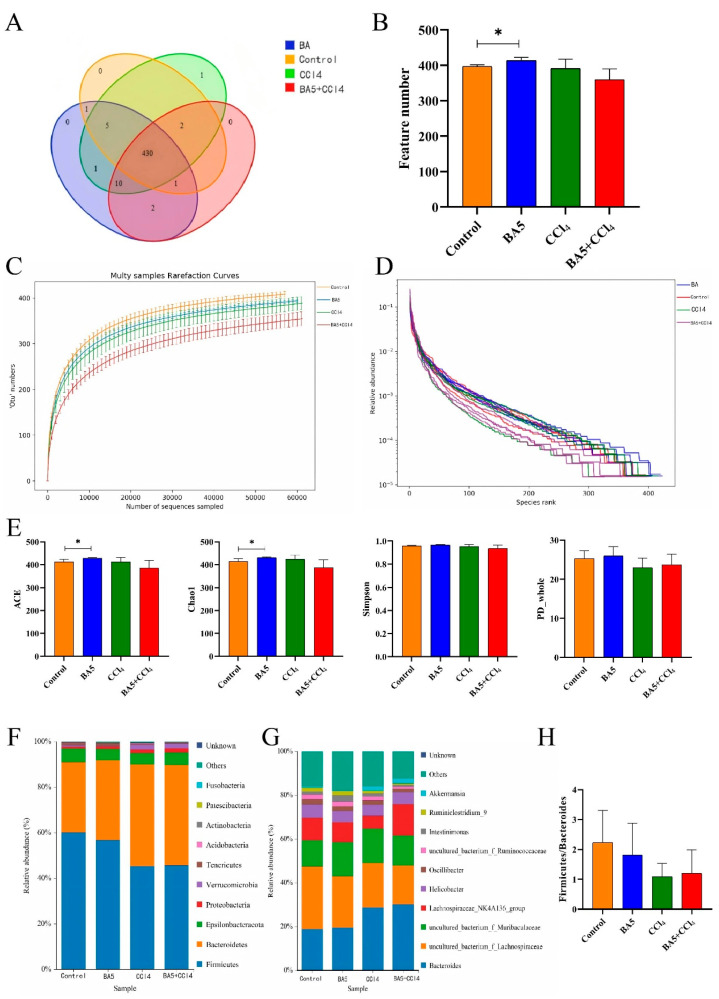
Effects of *B. amyloliquefaciens* BA5 on gut microbial diversity in CCl4-induced mice. (**A**) Venn diagram; (**B**) OTU numbers in samples. (**C**) Rarefaction curve. (**D**) Rank–abundance curve. (**E**) Alpha-diversity indices. (**F**) The predominant bacteria at phylum and (**G**) genus levels. (**H**) Firmicutes-to-Bacteroidetes (F/B) ratio in the gut microbiota of the Control, BA5, CCl_4_, and BA5 + CCl_4_ groups Data was expressed as the mean ± SD (*n* = 4 biological replicates randomly selected from each group; * *p* < 0.05).

Rank-abundance and rarefaction curves were used to evaluate whether the sequencing depth was sufficient for each sample. Both curves reached a plateau, indicating adequate sequencing depth (Figure 4C,D). To explore the impacts of BA5 on CCl_4_-induced intestinal diversity and composition in mice, 16 cecum contents were amplified and sequenced in the current study, and a total of 1,279,569 (Control = 319,853, BA5 = 320,305, CCl_4_ = 320,089, BA5 + CCl_4_ = 319,322) raw sequences were obtained, after which 1,222,575 (Control = 306,767, BA5 = 306,434, CCl_4_ = 305,415, BA5 + CCl_4_ = 303,959) effective reads were acquired after quality control, with an efficiency of greater than 90% per sample (Table 2). Subsequently, these valid reads were clustered into 453 OTUs (Figure 4B) and there were 430 common OTUs in all groups, accounting for 94.92% of the identified OTUs (Figure 4A).

Moreover, alpha diversity indices were also calculated, which reflected microbial diversity and richness. There was a distinct difference (*p* < 0.05) in ACE (412.915 ± 10.027 versus 427.695 ± 3.312, *p* < 0.05) and Chao1 (413.492 ± 12.373 versus 430.786 ± 3.153, *p* < 0.05) between the Control and BA5 groups, indicating that BA5 could enhance gut microbial diversity (Figure 4E).

Figure 4F illustrates the proportion of relative abundance occupied by microorganisms. Among identified phyla, the top five in abundance are *Firmicutes* (60.12%, 56.78%, 45.34%, and 45.68%), *Bacteroidetes* (30.85%, 35.02%, 44.69%, and 44.04%), *Epsilonbacteraeota* (6.02%, 5.02%, 4.99%, and 5.52%), *Proteobacteria* (0.77%, 1.12%,1.53%, and 1.82%), and *Verrucomicrobia* (0.63%, 0.24%, 1.96%, and 2.11%) in the Control group, BA5 group, CCl_4_ group, and BA5 + CCl_4_ group, accounting for more than 90% of the total microbial composition. Other phyla such as *Tenericutes*, *Acidobacteria*, *Actinobacteria*, *Patescibacteria*, and *Fusobacteria* were detected at low richness in all groups. At the genus level (Figure 4G), the five most abundant taxa were *Bacteroides* (18.88%, 19.54%, 28.79%, and 30.21%), *uncultured_bacterium_f_Lachnospiraceae* (28.59%, 23.49%, 20.21%, and 17.74%), *uncultured_bacterium_f_Muribaculaceae* (11.94%, 15.43%, 15.71%, and 13.69%), *Lachnospiraceae_NK4A136_group* (10.32%, 9.29%, 5.92%, and 14.26%), and *Helicobacter* (6.02%, 5.02%, 4.99%, and 5.52%) in the Control, BA5, CCl_4_, and BA5 + CCl_4_ groups, respectively, collectively accounting for approximately 80% of the total microbial composition. However, other genera such as *Oscillibacter*, *uncultured_bacterium_f_Ruminococcaceae*, *Intestinimonas*, *Ruminiclostridium_9*, and *Akkermansia* accounted for a low abundance in all groups. Firmicutes and Bacteroides occupied a dominant position in the intestinal tract, and their quantity and proportion were related to the health of the intestinal tract, thus F/B ratio was an important indicator reflecting the microecology of the intestinal tract. In the current study, the F/B ratio in the CCl_4_ group was reduced when compared with the Control group, while the ratio increased in the BA5 + CCl_4_ group as shown in Figure 4H.

### 3.5. BA5 Altered Gut Microbial Composition in CCl_4_-Induced Mice

**Figure 5:**
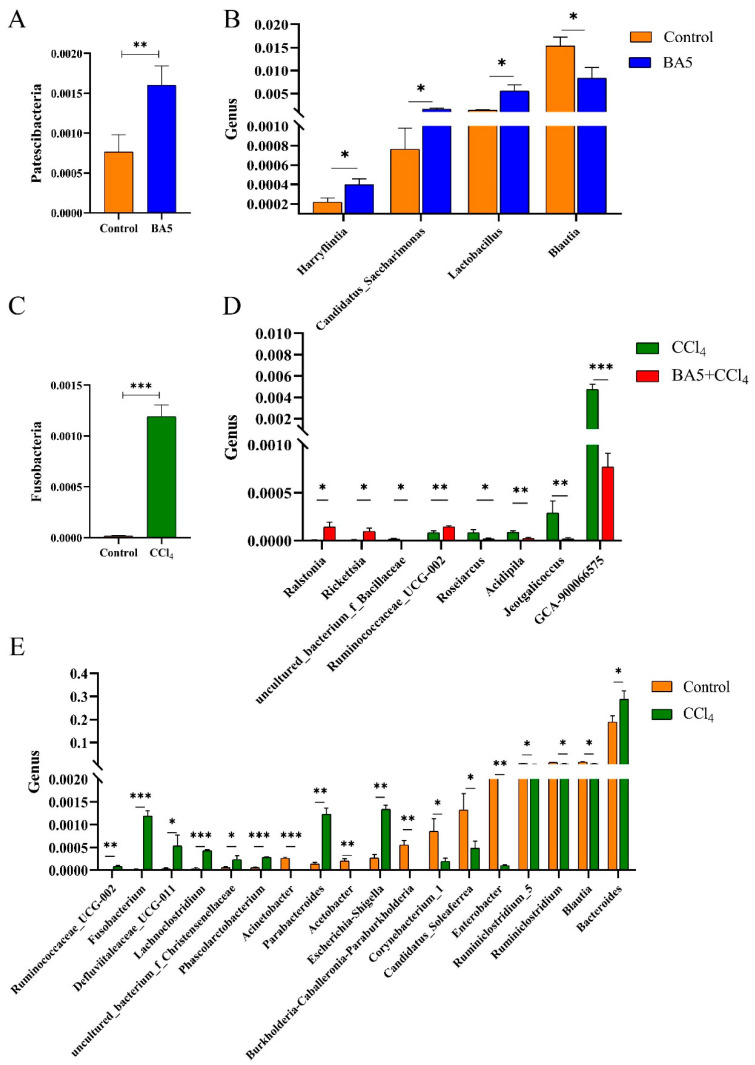
Effects of *B. amyloliquefaciens* BA5 on gut microbial composition in CCl_4_-induced mice. (**A**) The abundance of Patescibacteria at the phylum level between Control and BA5 groups. (**B**) Remarkable changes in the gut bacterial abundance at the genus level between Control and BA5 groups. (**C**) The abundance of Fusobacteria at the phylum level between Control and CCl_4_ groups. (**D**) Remarkable changes in the gut bacterial abundance at the genus level between CCl_4_ and BA5 + CCl_4_ groups. (**E**) Remarkable changes in the gut bacterial abundance at the genus level between Control and CCl_4_ groups. Data was expressed as the mean ± SD (*n* = 4 biological replicates randomly selected from each group; * *p* < 0.05; ** *p* < 0.01; *** *p* < 0.001).

**Figure 6:**
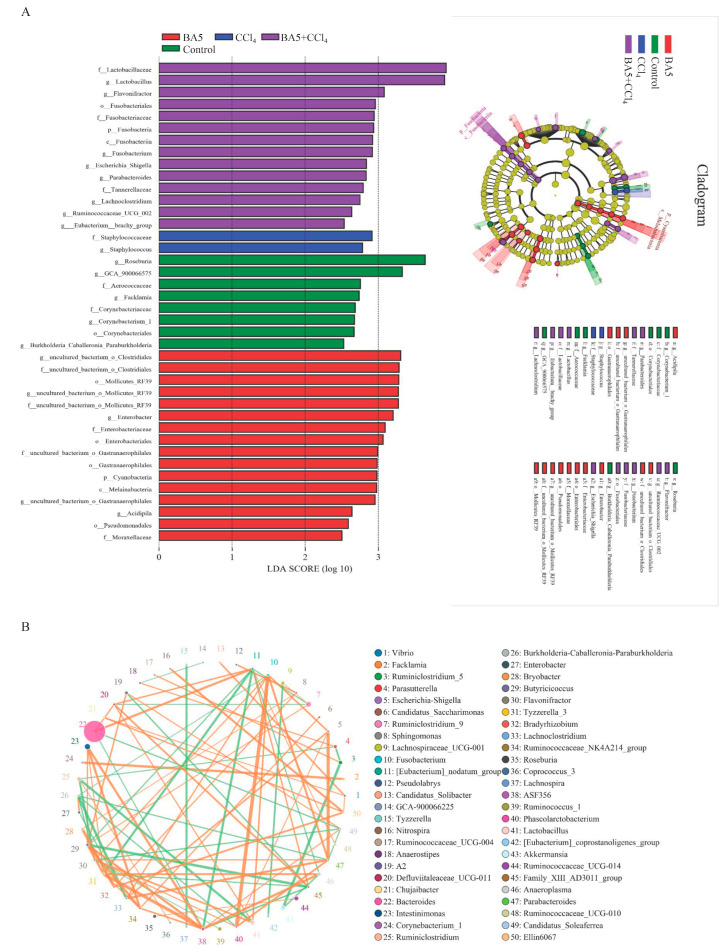
LEfSe analysis and correlation network analysis among different bacteria. (**A**) LEfSe analysis. (**B**) Correlation network analysis. Data was expressed as the mean ± SD (*n* = 4 biological replicates randomly selected from each group).

At the phylum level (Figure 5A), the relative abundance of *Patescibacteria* was significantly higher in the BA5 group compared with the Control group (*p* < 0.01). Conversely, *Fusobacteria* exhibited a markedly higher abundance in the CCl_4_ group relative to the Control (*p* < 0.001) (Figure 5C). At the genus level, 50 genera were detected to be obviously different between samples. Among them, compared with the Control group, the relative abundances of four bacterial genera (*Harryflintia*, *Candidatus_Saccharimonas*, and *Lactobacillus*) were markedly increased in the BA5 group (*p* < 0.05), whereas the relative abundance of *Blautia* was significantly reduced in the BA5 group (*p* < 0.05) (Figure 5B). As shown in Figure 5E, the relative abundances of nine genera (*Lachnoclostridium*, *Phascolarctobacterium*, *Fusobacterium*, *Escherichia–Shigella*, *Parabacteroides*, *Ruminococcaceae_UCG-002*, *Bacteroides*, *uncultured_bacterium_f_Christensenellaceae*, and *Defluviitaleaceae_UCG-011*) in the CCl_4_ group were investigated to be significantly greater when compared with the Control group (*p* < 0.05 or 0.01 or 0.001), while the relative abundances of nine genera (*Acinetobacter*, *Burkholderia-Caballeronia-Paraburkholderia*, *Enterobacter*, *Acetobacter*, *Blautia*, *Corynebacterium_1*, and *Candidatus_Soleaferrea*) were distinctly decreased (*p* < 0.05 or 0.01). Furthermore, the relative abundance of three genera (*Ruminococcaceae_UCG-002*, *Ralstonia*, and *Rickettsia*) in the BA5 + CCl_4_ group were significantly increased compared with the CCl_4_ group (*p* < 0.05 or 0.01) while the relative abundance of five genera (*Acidipila*, *Roseiarcus*, *uncultured_bacterium_f_Bacillaceae*, *Jeotgalicoccus*, and *GCA-900066575*) in the BA5 + CCl_4_ group was markedly decreased than the CCl_4_ group (*p* < 0.05 or 0.01 or 0.001) (Figure 5D). Additionally, LEfSe analysis combined with LDA score evaluation revealed distinct microbial biomarkers across treatment groups. In the Control group, *Roseburia*, *GCA-900066575*, *Facklamia*, and *Corynebacterium_1* were identified as dominant genera. *Staphylococcus* was predominant in the CCl_4_ group, whereas *Lactobacillus* emerged as the key dominant genus in the BA5 + CCl_4_ group (Figure 6A).

### 3.6. Correlation Network Analysis of Bacterial Community Interactions

Using Python 3.12 to construct the species correlation network, Figure 6B displays the top 50 genera with the strongest correlations. Each circle represents a genus, with its size indicating the average relative abundance. Lines between circles represent pairwise correlations, whereas line thickness reflects the strength of the correlation. Moreover, orange lines represent positive correlation and green lines represent negative correlation. The results indicated that *Lactobacillus* was positively related to *Fusobacteria* (0.8118), *Defluviitaleaceae_UCG-011* (0.7824), and *Flavonifractor* (0.8206) and was negatively related to *Ruminiclostridium* (0.8588). In addition, *Bacteroides* was positively related to *[Eubacterium]_brachy_group* (0.8529) and *Defluviitaleaceae_UCG-011* (0.7971), but was negatively related to *ASF356* (0.7706) and *Candidatus_Soleaferrea* (0.7824). *Blautia* was positively associated with *Ruminiclostridium* (0.5176), *Roseburia* (0.5676), *Lachnospiraceae_UCG-001* (0.5441), *Defluviitaleaceae_UCG-011* (0.7294), *Candidatus_Soleaferrea* (0.5059), *Enterorhabdus* (0.5118), and *Dorea* (0.6141), but was negatively related to *[Eubacterium]_brachy_group* (0.5588), *Erysipelatoclostridium* (0.5176), *Flavonifractor* (0.5353), *Fusobacterium* (0.5), *Defluviitaleaceae_UCG-011* (0.7294), *Anaerostipes* (0.5119), and *Lachnoclostridium* (0.543). Moreover, *Butyricicoccus* was positively related to *Anaerotruncus* (0.6265), *Ruminiclostridium_6* (0.5676), *Candidatus_Soleaferrea* (0.6294), *Enterorhabdus* (0.5176), and *Dorea* (0.5772), but was negatively associated with *Lachnospiraceae_FCS020_group* (0.5382), *Ruminococcaceae_UCG-010* (0.5206), *Escherichia–Shigella* (0.5), *Candidatus_Stoquefichus* (0.5353), and *Fusobacterium* (0.5).

## 4. Discussion

Over the last few years, liver diseases have been on the rise, resulting in them being major causes of morbidity and mortality across the world. It is worth noting that liver cancer is one of the deadliest forms of cancer, and currently, it is the second most common cancer in China [33,34]. Acute liver injury is acknowledged as one of the characteristic pathological alterations of different liver diseases and is mainly caused by oxidative stress, inflammation, and apoptosis due to the excessive production of reactive oxygen species (ROS) [32,35]. *Bacillus amyloliquefaciens* BA5 was found to have a high anti-oxidant capacity, thus attracting therapeutic interest in liver injury associated with oxidative stress [24]. The mechanisms underlying the protective roles of *Bacillus amyloliquefaciens* BA5, however, in the prevention of liver pathology, especially via the alteration of the gut microbiota, are not well understood. Carbon tetrachloride (CCl_4_) has nevertheless remained a well-established and extensively utilized model to assess the hepatoprotective potential of candidate compounds in vivo [36,37]. Thus, the objective of the current study was to determine the protective property of *Bacillus amyloliquefaciens* BA5 on CCl_4_-induced acute liver injury and more importantly the relationship between *Bacillus amyloliquefaciens* BA5 and the gut microbiota. The results showed that pretreatment with BA5 had a significant effect in reducing liver damage through the improvement of anti-oxidant capacity as well as aiding in restoring gut microbial homeostasis in mice with acute liver injury.

In terms of modeling approach, previous studies were proven to be justified [32,35,38,39,40]. To assess the growth rate of the mice during the experimental period, body weight was recorded daily. No significant differences in body weight were observed between groups, which contrasts with the findings reported by Zhang et al. [41] Certain studies have indicated that weight loss was seen in CCl_4_-induced mice, whereas we found no significant changes in the weight of groups. We speculate that this difference can be explained by species-specific reactions and by the relatively short time period of our modeling. Specifically, KM mice are also characterized by rapid growth and high fertility, which possibly compensated for any possible weight differences. Other supplementary indicators in determining the physiological condition of the mice were body weight in addition to organ indices [42,43]. In the present study, the liver index was significantly higher in the CCl_4_ group compared with the Control group. However, the BA5 + CCl_4_ group exhibited a smaller increase, which was associated with attenuated liver damage. These findings are consistent with the results reported by Keshavarz et al. [44]. Furthermore, CCl_4_ treatment caused severe hepatocellular necrosis accompanied by inflammatory infiltration, while the degree of pathological damage was reduced in the pretreated BA5 group, and meanwhile, the BA5 prevention group exhibited intact and clear jejunal villi relative to the CCl_4_ group, both of which indicated that BA5 played a positive role in preventing liver and jejunum injury caused by CCl_4_. However, this study lacked as assessment of LPS permeability, which would have better elucidated the connection between the liver and the gut.

Alanine aminotransferase (ALT) and aspartate aminotransferase (AST) are released from hepatocytes into the bloodstream during liver injury. This occurs as cell membrane permeability increases, resulting in significantly elevated serum levels of AST and ALT [45,46,47]. Hence, serum levels of ALT and AST serve as essential biomarkers for assessing the extent of liver injury [48,49]. In the present study, serum AST and ALT levels were significantly elevated in the CCl_4_-induced group compared with the Control group. Conversely, BA5 treatment reversed these upward trends, indicating that BA5 pretreatment protects against injury caused by CCl_4_ exposure. These results are consistent with Chen et al. [38] who presented that probiotics exerted protective effects on improving CCl_4_-induced acute liver injury.

A range of anti-oxidant enzymes of endogenous origin (SOD, CAT, GPX, GR, and GST) are effective at preventing and neutralizing free radical-induced damage [50]. Malondialdehyde (MDA) is one of the products of lipid peroxidation, and its decreased levels suggest cellular damage and reduced anti-oxidant capacity [51]. Total anti-oxidant capacity (T-AOC) summarizes the total amount of enzymatic and non-enzymatic anti-oxidants in the body, which is a complete measure of the body’s capacity to overcome oxidative stress and to remove the damage caused by free radicals specifically [52]. The current findings indicated that BA5 pretreatment greatly lowered the high serum MDA and reinstated the low levels of SOD, GSH-Px, and T-AOC caused by exposure to CCl_4_. The results are in line with previous findings that reported the strong anti-oxidant potential of *Bacillus amyloliquefaciens* [53,54].

Past research demonstrated that the Nrf2/HO-1 signaling pathway is one of the key factors involved in overall control over oxidative stress and healthy cellular living by maintaining redox homeostasis [55]. When oxidation occurs, Nrf-2 transfers from cytoplasm to the nucleus, which results in the expression of anti-oxidant enzymes (HO-1, NQO1, and GCLC) that reduce oxidation levels [56]. It has been shown in earlier research that pine nut polysaccharides have hepato-protective properties in carbon tetrachloride-induced hepatic injury in mice and its mechanism mainly relies on the Nrf2/ARE signaling pathway [57]. In the present study, CCl_4_ markedly inhibited the expression of the Keap1/Nrf-2 pathway in the liver; however, BA5 reverted the decreasing trend via elevating the expression of anti-oxidant enzymes (HO-1, NQO1, and GCLC), which was in line with the results of Chen [56]. The results suggested that BA5 improved the anti-oxidant capabilities in CCl_4_-induced mice.

The gut microbiota dynamically evolves with their hosts and is an integral part of the body, with various essential functions in the immune, metabolic, structural, and neurological landscapes of the body [58]. Under normal conditions, the composition and abundance of gut microbiota remain relatively stable. However, changes in the host’s internal or external environment can disrupt this balance, typically resulting in a decrease in beneficial bacteria and an increase in pathogenic or opportunistic microbes [59]. Accumulating evidence suggests that the toxic effects of CCl_4_ involve the gut microbiota [36,60]. Furthermore, numerous studies have illustrated that gut microbiota dysbiosis is closely associated with acute liver disease [61,62]. While various polysaccharides, *Lactobacillus strains*, and traditional medicines have been shown to protect against CCl_4_-induced acute liver injury in mice by modulating the gut microbiota, the effects of *Bacillus amyloliquefaciens* remain poorly described [32,38,57,63,64]. To examine the consequences of CCl_4_ exposure and BA5 intervention on microbial diversity in the gut, a high-throughput 16S rRNA gene sequencing technique was used as a measure of the relative gene abundance and representation of the microorganisms per group. The richness and evenness of microbial species within a single sample (alpha diversity) was assessed using several indices such as Chao1, ACE, Shannon’s, Simpson’s, and PD whole tree. Also in this study, species richness increased in the BA5-treated gut environment more than in the Control group, which indicated that the BA5-treated gut environment was more conducive to microbial colonization and survival. Interestingly, the PD whole tree index, a parameter of phylogenetic diversity had a lower value in the CCl_4_-treated group compared with the Control group, which showed a loss in the phylogenetic complexity. This downward trend was reversed in the BA5-treated group; however, the change was not statistically significant, likely due to the small sample size. Moreover, we examined the compositional makeup of the gut microbiota and specifically Firmicutes/Bacteroidetes ratio (F/B). Shifts in the ratio of F/B are generally accepted as signs of microbial maladaptation within the gut and changes in the balance were linked to multiple diseases [65,66].

In order to investigate the variation in microbial community abundance between the two groups of samples, a T-test was performed on the species abundance data between the groups using Metastats software [67]. At the phylum level, *Patescibacteria* and *Fusobacteria* were dominant bacteria in the BA5 and CCl_4_ groups, respectively. *Patescibacteria*, also known as the candidate phyla radiation (CPR), are a diverse group of bacteria [68]. *Fusobacteria* are commensal organisms in humans and animals; however, certain species act as opportunistic pathogens, capable of causing bacteremia and various rapidly progressing infections. At the genus level, an increase in potentially harmful intestinal bacteria such as *Fusobacterium*, *Lachnoclostridium*, *Phascolarctobacterium*, and *Escherichia–Shigella* was observed in the CCl_4_ group. In contrast, beneficial bacteria such as Blautia, a newly recognized functional genus with potential probiotic properties, were significantly reduced [69]. *Fusobacterium* has long been found to cause opportunistic infections and are related to colorectal cancer [70]. A previous study has suggested that *Lachnoclostridium* were identified to be significantly enriched in colorectal adenoma and cancer [71]. Bucher-Johannessen C. et al. [72] had verified that *Phascolarctobacterium* were increased in adenoma and colorectal cancer (CRC) and this species should be recognized among the most important CRC-associated bacteria. *Escherichia–shigella* and enteroinvasive *Escherichia coli* (EIEC) are Gram-negative bacteria responsible for human bacillary dysentery (shigellosis) and are characterized by the invasion and destruction of the human colonic epithelium [73]. Moreover, there were three bacteria (*Harryflintia*, *Candidatus_Saccharimonas*, and *Lactobacillus*) that were dramatically increased and one bacterium (*Blautia*) was significantly decreased in the BA5 group compared with the Control group. Notably, the LefSe results have shown that *Lactobacillus* also distinctly increased in the BA5 + CCl_4_ group, indicating that BA5 treatment improved the abundance of *Lactobacillus* to resist the toxicity from CCl_4_, which was in line with Chen’s study [38]. In addition, previous studies have emphasized that *Bacillus amyloliquefaciens* inhibited cecal inflammation in mice by regulating intestinal flora [18]. On the basis of the obtained results. Therefore, we hypothesize that BA5 may alleviate CCl_4_-induced intestinal damage, through its effects on microbial richness and gut microbial composition by changing the relative abundance of bacterial taxa, particularly beneficial ones like *Lactobacillus*. Such gut microbiota modulation can potentially lead to the normalization in gut–liver axis homeostasis, and therefore, protect liver functionality. However, two primary limitations of the current study warrant acknowledgement. First, regarding the experimental model, although carbon tetrachloride is a prototypical xenobiotic for inducing acute hepatotoxicity and fibrosis, it represents a chemical injury paradigm that does not fully recapitulate the complex etiology and metabolic dysregulation inherent to prevalent human liver diseases. Second, the precise molecular mechanisms underlying the hepatoprotective efficacy of BA5 remain to be fully elucidated. Therefore, future research needs to validate the hepatoprotective effects of *B. amyloliquefaciens* BA5 at the molecular level in more physiologically relevant models (such as high-fat diet or alcohol-induced liver injury models) to fully confirm its potential for clinical translation.

## 5. Conclusions

The present study concludes that *Bacillus amyloliquefaciens* BA5 exerts hepatoprotective effects by alleviating CCl_4_-induced acute liver injury and oxidative stress. Furthermore, BA5 alleviated CCl_4_-induced intestinal dysfunction and damage by reducing the proportions of pathogenic taxa, such as *Fusobacterium*, *Lachnoclostridium*, *Phascolarctobacterium*, and *Escherichia–Shigella*, while increasing the abundance of beneficial *Lactobacillus*. However, further studies are required to elucidate the potential mechanisms underlying the gut–liver axis in this model.

## Figures and Tables

**Table 1:** Primer sequence information of target genes.

Gene	Primer Sequence
Nrf-2 (F)	CACCCATGACTCATTTAAGCAC
Nrf-2 (R)	CACCTGCTTCTTTTGGCTATTA
HO-1 (F)	TCCTTGTAGGATATCTACACCC
HO-1 (R)	GACACGCTTTACATAGTCCTGT
NQO1 (F)	GAAGACATCATTCAACTACGCC
NQO1 (R)	GAGATGACTCGGAAGGATACTG
GCLC (F)	CTATCTGCGGAATTGTTATCCC
GCLC (R)	CCTCGGGTCTTCTATCATCTAC
β-ACTIN (F)	CCTAGACTTCGAGCAAGAGA
β-ACTIN (R)	GGATGGAAGCCTGGATGT

**Table 2:** Quality assessment of sequencing data.

Group	Control	BA5	CCl_4_	BA5 + CCl_4_
Raw Reads	79,963 ± 257	80,076 ± 53	80,022 ± 259	79,830 ± 389
Effective Reads	76,692 ± 629	76,608 ± 399	76,354 ± 659	75,990 ± 935
Mean length (bp)	411 ± 2	413 ± 2	415 ± 2	415 ± 3

## Data Availability

The raw sequencing data for the 16S rRNA gene sequence are available on NCBI under Submission ID: PRJNA1053417, https://www.ncbi.nlm.nih.gov/bioproject/PRJNA1053417/, accessed on 15 December 2023.
